# Combined multichannel intraluminal impedance and pH testing in infants and young children—a narrative review

**DOI:** 10.3389/fped.2025.1675149

**Published:** 2025-10-01

**Authors:** Rochelle Sequeira Gomes, Sheeja Abraham, Michael T. Favara, Amy J. Sloane, Zubair H. Aghai

**Affiliations:** ^1^ Division of Neonatology, Nemours Children’s Health, Orlando, FL, United States; ^2^ Division of Gastroenterology, Nemours Children’s Health, Philadelphia, PA, United States; ^3^ Division of Neonatology, ChristianaCare, Newark, DE, United States; ^4^ Division of Neonatology, Nemours Children’s Health at Thomas Jefferson University Hospital, Philadelphia, PA, United States

**Keywords:** combined multichannel intraluminal impedance and pH testing, gastroesophageal reflux - diagnosis, treatment, GERD in children, GERD symptoms in children, GERD phenotypes

## Abstract

Gastroesophageal reflux disease (GERD) is common in infants and young children, and thediagnosis and characterization of the disease continues to evolve in recent times. The typicalsymptoms thought to be related to GERD in young children are often nonspecific andubiquitous, underscoring the need for diagnostic testing in patients who have severe symptomsor complications related to GERD. Combined multichannel intraluminal impedance and pHtesting (MII-pH) is a diagnostic tool that can be used to study the frequency andseverity of gastroesophageal reflux. Compared to other diagnostic devices, MII-pH hasthe advantage of detecting both acidic and alkaline reflux events, as well as allows the study of thetemporal association of symptoms with GER events. Using the diagnostic parameters definedand symptom association data obtained, MII-pH can then be used to classify patients as havingGERD (with either predominantly acid or alkaline reflux), non-erosive reflux disease or NERD (when noevidence of esophagitis is noted on endoscopy), hypersensitive esophagus (positive symptom association only) and functional heartburn (normal study). The application of this disease classification to GERD in young children is relatively new and needs further validation. Even so, classifying GERD into these phenotypes using MII-pH allows for more precise and individualized therapeutic decisions. Emerging research has also suggested MII-pH testing can be used to predict changes in mucosal integrity and study the motility of the esophagus in children. Although the use of MII-pH in children with a large range of disease processes is becoming more widespread, there are important limitations to note in the interpretation of results of MII-pH in young children, due to the relative lack of normative data obtained from truly healthy children.

## 1 Background

Gastroesophageal reflux (GER), defined as “the passage of gastric contents into the esophagus with or without regurgitation and vomiting” is a common physiological occurrence in infants and young children due to decreased lower esophageal tone, small stomach capacity, delayed gastric emptying and supine positioning. It is considered pathological only when associated with troublesome symptoms that affect daily function and/or with complications (1). However, applying these diagnostic criteria for GER in this population is challenging due to the nonspecific, ubiquitous nature of reflux symptoms. Differentiating true gastroesophageal reflux disease (GERD) from physiological GER remains difficult due to considerable variation in the definitions and outcome measures used in the literature (2).

The use of symptomatology alone to diagnose and treat GERD is likely to lead to overdiagnosis and over treatment in young children. The American Academy of Pediatrics (AAP) advises against the routine use of anti-reflux medication for GER symptoms alone (3). Utilizing accessible, safe, and cost-effective means for diagnosing GERD, particularly in high-risk infants and young children, would facilitate the delivery of targeted therapy when clinically indicated. In 2018, the North American Society for Pediatric Gastroenterology, Hepatology, and Nutrition (NASPGHAN) and the European Society for Pediatric Gastroenterology, Hepatology, and Nutrition (ESPGHAN) issued updated guidelines on GERD management. Although there is no universal gold standard for GERD diagnosis in infants and young children, a step wise approach with screening for alarm symptoms, then dietary modifications, followed by a brief trial of acid suppression and referral to gastroenterology specialists as a “third line” for testing was recommended. Despite its advantages, it was concluded that there is currently insufficient evidence to support the use of combined multichannel intraluminal impedance and pH testing (MII-pH) as a standalone diagnostic tool for the diagnosis of GERD in infants and children (1). The working group suggested considering the use of MII-pH to correlate (a) persistent troublesome symptoms with acid and alkaline reflux events, (b) clarify the role of acid and alkaline reflux in the etiology of esophagitis, (c) determine the efficacy of acid suppression therapy, and (d) differentiate non-erosive reflux disease, hypersensitive esophagus and functional heartburn in patient with normal endoscopy (1). This review aims to summarize findings from existing literature involving cohorts of young children with and without GER symptoms, who have undergone MII-pH studies. Additionally, we also report expert consensus guidelines on the use of MII-pH for GER evaluation in this population, including how to interpret and apply the MII-pH results in different GER phenotypes. This review also addresses the clinical relevance of novel MII-pH indices and their potential application. Lastly, we highlight the current limitations in applying the results of MII-pH in young children, emphasizing the gaps in the literature and the need for further studies to demonstrate improved clinical outcomes with the use of MII-pH in this demographic.

## 2 Combined multichannel intraluminal impedance and pH testing (MII-pH): equipment and technique

Impedance, defined as voltage divided by current, is inversely proportional to the ionic concentration of a medium. It is measured in ohms. The use of impedance measurements to study GER was first described in 1991 (4), followed by guidance on pediatric application (5–8). The technique involves the use of an appropriately sized nasogastric catheter, typically equipped with six or seven impedance sensing electrodes or rings along its length. There are usually 6 impedance channels, one channel being the segment between two adjacent electrodes. As esophageal contents move along the length of the catheter, the impedance electrodes sense the change in electrical impedance, thereby determining the nature (gas vs. liquid) and location (upper vs. lower esophagus) of the refluxate. Additionally, one distal electrode also carries a pH sensor made of antimony, ion-sensitive field-effect transistor (ISFET), or glass. Catheters may also incorporate a feeding tube or a simplified manometric device. The diameter of the probe is typically 2.13 mm (6.4 Fr) and both single use and multiuse catheters are available, with either internal (preferred) or external (skin) reference electrodes. Ambulatory device set ups are also available. Catheter size selection is based on the patient's height or age, as outlined in Table 1 (7, 8).

**Table 1:** Catheter type for patient height and age.

Catheter type	Patient height	Patient age
Infant catheter	<75 cm	Birth – 2 years
Pediatric catheter	75–150 cm	2–10 years
Adult catheter	>150 cm	>10 years

**Figure 1:**
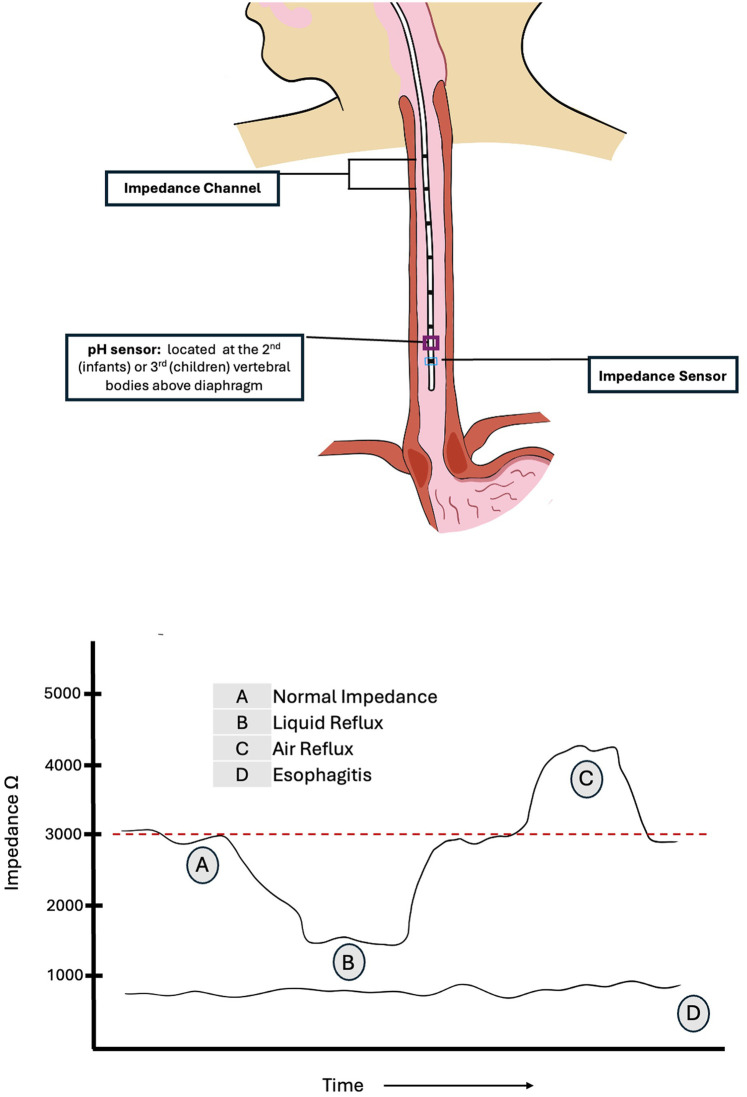
MII-pH catheter placement in children and impedance interpretation.

Prior to each use, pH calibration is performed using liquids with two standard pH checks (typically pH 4 and 7), as recommended by the manufacturer. Each impedance electrode should also be tested to confirm conductivity and integrity. If a lubricant is used for insertion, care should be taken to avoid gel being placed on the antimony electrode, as this could interfere with accurate measurements. Placement depth in infants can be estimated using the Strobel formula [0.252 × height (cm) + 5] (9), although this formula may overestimate the depth of insertion in children older than one year of age. Two novel approaches – the Great Ormond Street Hospital (GOSH Table) and the KidZ Health Castle formula (KHC-F) – have been recently introduced to better aid in determining MII-pH catheter length, yielding reliable results (10–12). Placement of the end of the catheter is generally guided by the position of the pH sensor. For catheters used in infants and children, the pH sensor is located in the middle of the most distal impedance channel, 0.75–1 cm above the most distal impedance probe. Optimum placement of the pH sensor for infants and newborns is generally lower, at the second vertebral body above the diaphragm, to avoid discomfort associated with proximal pharyngeal extension of the catheter as well as artifacts in the tracing. For older children however, the tip may be positioned higher, at 3 vertebral bodies above the diaphragm throughout the respiratory cycle, to avoid proximity to the gastro-esophageal junction (6, 8, 13). Figure 1 illustrates the impedance channels and pH sensor on the catheter and appropriate placement in children with interpretation of impedance changes. MII-pH monitoring usually lasts for 18–24 h. To capture the desired information, the study should be carried out at baseline conditions, as factors such as diet, feeding tubes, physical exertion and body position may affect the occurrence of reflux. The catheter is placed following a short, age appropriate, period of fasting (typically 2–3 h), and the study is then commenced.

Baseline impedance generally ranges from 2,000 to 4,000 ohms (8). However, a study in asymptomatic preterm infants demonstrated that basal impedance is lower in very young infants, with a median value of 1,750 ohms [IQR: 1,500–2,050] (14). By convention, during the MII-pH study, a successive drop in impedance by more than 50% from baseline observed in at least two distal electrodes in retrograde direction from the stomach is recorded as a liquid reflux event. It is important to note that certain pathological conditions such as esophagitis or motility disorders may be associated with lower baseline impedance in the esophagus, which can make it more difficult to detect a 50% further drop in impedance. Consequently, this may result in lower rates of reflux detection when using MII-pH in these conditions (6, 15, 16). Baseline impedance values below 900 ohms have been shown to have a 100% positive predictive value for severe esophagitis in children (17). Additionally, lower esophageal baseline impedance levels have been observed in other GER-associated comorbidities such as bronchopulmonary dysplasia of prematurity (18). Impedance testing can also differentiate between air, which produces a rise in impedance, typically above 3,000 ohms, and liquid which produces a drop in impedance. These changes are often represented in color-coded plot.

The pH sensing electrode allows for the detection of acidic (pH < 4 for at least 5 s), weakly acidic (pH 4–7) and alkaline (pH > 7) reflux. Acid reflux (AGER) is identified by changes in both impedance and pH, whereas non-acidic GER (NAGER) is detected solely by change in impedance. “pH only” events (POEs) characterized by a drop in pH without a corresponding change in impedance, may reflect acid exposure in the esophagus unrelated to reflux, such as that caused by feeding, swallowing, or delayed clearance of prior refluxate. POEs are reported to occur frequently in infants and may also represent short column or low volume acidic reflux (7, 14).

Observation of the patient and time marking the occurrence of GER symptoms using the event button on the device or writing it in a diary can facilitate temporal correlation of symptoms with reflux events. Similarly, time marking the start and end of enteral feeds can also help exclude feeding periods from data analysis. Commercially available impedance devices provide software that graphically displays study results, illustrating the timing, height, duration, and pH of reflux events. Additionally, the software can compute indices commonly used to quantify symptom association, as described below. The accuracy of symptom reporting is heavily dependent on the consistency and reliability of the person recording symptom events, which has been shown to be variable (19, 20). The diagnostic yield of symptom indices may be enhanced using video monitoring during MII-pH studies (21).

## 3 Diagnostic parameters in MII-pH

Table 2 illustrates the diagnostic parameters reported in MII-pH studies with values reported in different cohorts of symptomatic and non-symptomatic young children. Abnormal cut-off points in infants and young children are somewhat arbitrary and difficult to validate in large studies, due to the challenges with carrying out this long and expensive study in asymptomatic children. There is wide variation in the reported results of these diagnostic parameters, with significant overlap in values between symptomatic and asymptomatic children. Hence, it is preferable to use multiple parameters, including the symptom association indices described below, to define disease phenotypes and make management decisions.

**Table 2:** Diagnostic parameters in MII-pH.

Diagnostic parameter	Definition	Subjects: Values Median (95^th^ centile) or (IQR) unless specified	Age, number of subjects Median (IQR) or (range) or mean ± SD	Ref
Total number of reflux events (also referred to as retrograde bolus movements or RBM)	Number of acid and non-acid reflux events during study period (calculated for 24 h)	Asymptomatic preterm neonates: 71 (100.7)	12 days (IQR 9–17.5 days), *n* = 21	(14)
		*Symptomatic children with “normal” MII-pH study Newborns: 72.24 (136.08) Infants: 65.76 (117.12) Children: 50.16 (105.6)	18 days (3–30 days), *n* = 46 64 mo (32–328 mo), *n* = 83 5 yr (1–16 years), *n* = 66	(26)
		Symptomatic preterm infants: 44 (103) Symptomatic term infants: 52 (107)	71 days (IQR 44–114 days), *n* = 119 44 days (IQR 28–71 days), *n* = 62	(24)
		Infants without GERD: 54 (93) Children without GERD:21 (71)	4.8 mo (range 3 wks–11.9 mo), *n* = 46 7.2 years (range 1.3–17 years), *n* = 71	(28)
		Infants with chronic cough: 110 (84–140.3) Children with chronic cough: 110.5 (72–146)	5 mo (3–7 mo), *n* = 168 36 mo (17–64.5 mo), *n* = 258	(30)
		Symptomatic infants: Mean 43 (1–136) Symptomatic children: Mean 41.9 (0–238)	<1 years, *n* = 83 >1 years, *n* = 282	(29)
		Control children: 31 (69) Children with GERD: 51 (98)	7.8 years ± 6.8, *n* = 10 6.2 years ± 3.8, *n* = 10	(27)
		Symptomatic children Group 1: 64 (range 11–185) Group 2: 34 (range 2–169) Group 3: 27 (range 0–213)	0–12 mo (*n* = 116) 1–2 years (*n* = 53) 2–5 years (*n* = 114)	(19)
Number of acid reflux events or AGER	Number of acid reflux (pH < 4)	Symptomatic preterm infants: 14 (39) Symptomatic term infants: 17 (40)	71 days (IQR 44–114 days), *n* = 119 44 days (IQR 28–71 days), *n* = 62	(24)
		Control children: 24.5 (58) Children with GERD: 47 (83)	7.8 years ± 6.8, *n* = 10 6.2 years ± 3.8, *n* = 10	(27)
		Infants without GERD: 20 (48) Children without GERD: 14 (55)	4.8 mo (range 3 wks–11.9 mo), *n* = 46 7.2 years (range 1.3–17 years), *n* = 71	(28)
Percentage of acid reflux events	Percent of events with pH < 4 in study period	Asymptomatic preterm neonates: 25.4% (52.3)	12 days (IQR 9–17.5 days), *n* = 21	(14)
		Infants with chronic cough: 19.9 (8.1–37.6) Children with chronic cough: 44.3(20.7–63.2)	5 mo (3–7 mo), *n* = 168 36 mo (17–64.5 mo), *n* = 258	(30)
Number of weakly acidic (pH 4–7) or alkaline (pH >7)reflux events or NAGER	Number of events with pH > 4	Symptomatic preterm infants: 24 (74) Symptomatic term infants: 25 (77)	71 days (IQR 44–114), *n* = 119 44 days (IQR 28–71), *n* = 62	(24)
		Control children: 8.5 (13) Children with GERD: 7 (15)	7.8 years ± 6.8, *n* = 10 6.2 years ± 3.8, *n* = 10	(27)
		Infants without GERD: 32 (67) Children without GERD: 6 (34)	4.8 mo (range 3 wks–11.9 mo), *n* = 46 7.2 years (range 1.3–17 years), *n* = 71	(28)
Reflux Index/(also referred to total acid exposure index)	Percentage of time with esophageal pH < 4.0	Asymptomatic preterm neonates: 5.59 (20.17)	12 days (IQR 9–17.5 days), *n* = 21	(14)
		Symptomatic preterm infants: 3 (15.87) Symptomatic term infants: 1.95 (8.88)	71 days (IQR 44–114 days), *n* = 119 44 days (IQR 28–71 days), *n* = 62	(24)
		Control children: 2.6 ± 1.9 Children with GERD: 11.3 ± 4.3	7.8 years ± 6.8, *n* = 10 6.2 years ± 3.8, *n* = 10	(27)
		Symptomatic children: 0.72 (IQR 0.04–3.41)	10.9 mo ± 7.2 mo, *n* = 20	(51)
		Symptomatic infants: Mean 4.2 (0.0–62.2) Symptomatic children: Mean 4.1 (0.0–68.2)	<1 years, *n* = 83 >1 years, *n* = 282	(29)
Number of episodes with reflux lasting >5 min		Symptomatic children: 0 (0–1)	10.9 mo ± 7.2 mo, *n* = 20	(51)
		Infants with chronic cough: 1 (0–4) Children with chronic cough: 3 (0–16)	5 mo (3–7 mo), *n* = 168 36 mo (17–64.5 mo), *n* = 258	(30)
Duration of longest episode	Maximum reflux time (minutes)	Infants with chronic cough: 6.55 (2.8–19.5) Children with chronic cough: 13.45 (4.0–59.8)	5 mo (3–7 mo), *n* = 168 36 mo (17–64.5 mo), *n* = 258	(30)
Acid reflux clearance time	Time for acid clearance (seconds)	Symptomatic infants: 171.6 (0–5,561) Symptomatic children: 172.6 (0–12,079)	<1 years, *n* = 83 >1 years, *n* = 282	(29)
Bolus clearance time or Bolus contact time	The time bolus stays in the distal esophagus (sec)	Symptomatic children with “normal” MII-pH study Newborns: 21.72 (33.43) Infants: 17.87 (39.47) Children: 16.00 (26.87)	18 days (3–30 days), *n* = 46 64 mo (32–328 mo), *n* = 83 5 yr (1–16 yr), *n* = 66	(26)
		Infants without GERD: 13 (20) Children without GERD: 15 (32)	4.8 mo (range 3 wks–11.9 mo), *n* = 46 7.2 yr (range 1.3–17 yr), *n* = 71	(28)
		Symptomatic infants: Median 12.0 (5–22) Symptomatic children: Median 17.4 (0–322)	<1 yr, *n* = 83 >1 yr, *n* = 282	(29)
		Symptomatic children Group 1: 14 (range 7–61) Group 2: 15 (range 8–41) Group 3: 17 (range 0–71)	0–12 mo (*n* = 116) 1–2 yr (*n* = 53) 2–5 yr (*n* = 114)	(19)
Bolus exposure index (BEI) %	The total percentage of time the bolus stays in the esophagus	Asymptomatic Neonates 0.73 (1.2)	12 days (IQR 9–17.5 days), *n* = 21	(14)
		Symptomatic children with “normal” MII-pH study Newborns: 2.03 (4.42) Infants: 1.50 (3.69) Children: 1.25 (2.73)	18 days (3–30 days), *n* = 46 64 mo (32–328 mo), *n* = 83 5 yr (1–16 yr), *n* = 66	(26)
		Control children: 0.89 ± 0.7 Children with GERD: 1.83 ± 0.77	7.8 yr ± 6.8, *n* = 10 6.2 yr ± 3.8, *n* = 10	(27)
% time AGER (excludes non reflux related acid)	The total percentage of time acid reflux is exposed in the esophagus	Asymptomatic Neonates: 1.66 (6.3)	12 days (IQR 9–17.5 days), *n* = 21	(14)
		Control children: 0.7 ± 0.56 Children with GERD: 1.63 ± 0.62	7.8 yr ± 6.8, *n* = 10 6.2 yr ± 3.8, *n* = 10	(27)
		Infants without GERD: 0.6 (1.4) Children without GERD: 0.4 (1.3)	4.8 mo (range 3 wks–11.9 mo), *n* = 46 7.2 yr (range 1.3–17 yr), *n* = 71	(28)
% time NAGER	The total percentage of time nonacid reflux is exposed in the esophagus	Control children: 0.14 ± 0.11 Children with GERD: 0.2 ± 0.37	7.8 yr ± 6.8, *n* = 10 6.2 yr ± 3.8, *n* = 10	(27)
		Infants without GERD: 0.7 (2.5) Children without GERD: 0.1 (1)	4.8 mo (range 3 wks–11.9 mo), *n* = 46 7.2 yr (range 1.3–17 yr), *n* = 71	(28)
PSPW Index % (Post-reflux swallow-induced peristaltic wave Index)	Number of PSPW events divided by the total number of reflux episodes	*GERD: 23.25 ± 23.87 NERD: 42.6 (29.6–45.8) *Reflux hypersensitivity: 47.7 ± 25.03 *Functional heartburn: 58.3 ± 41.34	2 mo to 17 yr, *n* = 479	(33, 38)
MNBI (ohms) (Mean nocturnal baseline impedance)	Mean of impedance values taken at 10-minute reflux free intervals during the night	*Gastroesophageal reflux disease: 1,253.7 ± 535.98 Non erosive reflux disease: 1315 (1018–2,832) *Reflux hypersensitivity: 1,794.5 ± 676.24 *Functional heartburn: 2,292.5 ± 552.63	2 mo to 17 yr, *n* = 479	(33, 38)

AGER, acid GER; NAGER, non-acidic GER; sec, seconds; mo, months; yr, years. All studies were a minimum of 18–20 h. Results are calculated for 24 h wherever applicable, to allow comparison.

* Computed values.

The reflux index (RI) and the number of reflux episodes (also known as retrograde bolus movements or RBM) per 24 h are the most frequently used parameters. The number of GER episodes can be reported as the total number of GER episodes, and the number and percentage of AGER and NAGER episodes. RI is defined as the percentage of time with esophageal pH < 4.0 in 24 h. RI often includes reflux and non-reflux related acid exposure. The threshold for abnormal RI in infants (<1 year) and children (>1 year) was recently recommended to be >10% and >5% respectively by some experts (1, 19), while in the past, others have recommended >12% and >6%, respectively (22). Previously, in 2009, both NASPGHAN and ESPGHAN had recommended that an RI threshold of <3%, 3%–7% and >7% be used as normal, indeterminate and abnormal, respectively (23). When compared to term infants and older healthy children, the RI was shown to be higher in both symptomatic and asymptomatic preterm infants (95th centile 15%–20% vs. ≤10% respectively) (14, 24, 25). The range of RI reported in children is wide, as shown in Table 2. Severe cohorts of children with GER symptoms are seen to have low/normal RI, which would suggest that measurement of esophageal acid exposure alone is not adequate to understand the pathogenesis of GER symptoms. It is also possible that in some patients, these purported GER symptoms may not be related to reflux at all.

Frequent reflux is also considered pathological if there are more than 100 GER events in 24 h in children less than one year of age and greater than 70 in those older than one year (6, 19). These thresholds have been validated in studies using MII-pH in symptomatic and asymptomatic infants <1 year, where it has shown that the 95th centile of number of reflux episodes is ∼100 episodes or higher per 24 h (14, 24, 26), whereas older children without GER symptoms have 70 events/24 h in the 95th centile (27, 28). Symptomatic older children with GER were commonly found to have >100 episodes in 24 h (29, 30).

From Table 2, approximately 25%–40% of reflux episodes are reported to be acidic in infants and young children, with the proportion of acid reflux episodes increasing with age. Prolonged reflux events greater than five minutes seem to be infrequent, although some outliers with prolonged reflux times are noted in some patients, possibly related to an associated esophageal motility disorder in those subjects.

Several authors also report time (in seconds or minutes) to clear acid reflux and/or bolus of refluxate and then calculate a clearance or exposure index as a percentage of exposure time to total study time. Some terms used to describe these are acid reflux clearance time, bolus clearance time or bolus contact time, and the percentage is expressed as the bolus exposure index (14, 19, 27, 29). As shown in Table 2, there is an inconsistent association between age and bolus clearance time, but this may be in part related to variable diets in different studies (solid vs. liquid). While faster bolus clearance in the younger age groups has been described (19, 28, 29), a recent study has shown the opposite relationship (26). In young infants and children, higher reflux and a higher bolus exposure index at initial diagnosis correlated with longer duration of symptoms (31). A recent small study showed a strong correlation between frequent and prolonged acid reflux exposure (duration of longest acid reflux ≥17 min, and occurrence of acid reflux for more than five minutes) detected by MII-pH with endoscopic evidence of significant esophagitis in children (32).

The mean nocturnal baseline impedance (MNBI) and post-reflux swallow-induced peristaltic wave (PSPW) index have recently been described in children with GER from two months to 17 years. The MNBI (the mean of impedance values taken at 10-min reflux free intervals during the night) is believed to reflect mucosal integrity and reflux burden, with a lower MNBI correlating with higher incidence of reflux esophagitis. The PSPW index (the number of PSPW events divided by the total number of reflux episodes) measures the ability of the esophagus to effectively clear refluxate following a GER episode and was shown to be inversely related to acid exposure time. These novel parameters may enhance the ability to differentiate various GER phenotypes in children using MII-pH (33).

## 4 Symptom association in MII-pH

While the reflux parameters described above are used to characterize the nature of reflux in terms of frequency, duration and pH, another important diagnostic consideration is the association of GER events with symptoms. The temporal association of symptoms with GER episodes can be determined with MII-pH testing by time marking the occurrence of symptoms during the study. Several authors have reported symptoms occurring both before and after reflux events, hence establishing association rather than causation. Variation exists in the time interval used to define positive association between symptom occurrence and GER episodes. Reported acceptable intervals range from 30 to 120 s (6, 19, 20) and may be as long as up to five minutes (24, 34–36). Additionally, some studies also suggest using different time intervals for different GER symptoms (6, 37). The three symptom indices - symptom index (SI), symptom sensitivity index (SSI) and symptom association probability (SAP) are then calculated as shown in Table 3. The symptom indices are measures of probability of the association of symptoms and GER episodes and are therefore influenced by the frequency of symptoms and GER events. Several authors have reported temporal symptom association data in cohorts of young children using MII-pH. Table 4 summarizes some of these recent studies. Symptoms studied include both gastrointestinal and non-gastrointestinal symptoms. As shown, variable proportions of symptomatic children in each cohort had positive symptom association, defined as SI > 50%, SSI > 10% and/or SAP > 95%.

**Table 3:** Indices used to quantify symptom association.

Index	Definition	Abnormal value	False positives
Symptom index (SI)	Percentage of symptoms temporally related to a GER episode	>50%	If high number of GER episodes and/or infrequent symptoms
Symptom sensitivity index (SSI)	Percentage of GER episodes that is temporally associated with symptoms	>10%	If the frequency of GER is low and/or frequent symptoms are present
Symptom association probability (SAP)	Statistical probability (using Fisher exact test) that symptoms and GER are unrelated. The *P*-value is subtracted from 100% to give the SAP	>95%	

**Table 4:** Symptom association using MII-pH in young children.

Indication for study	Sample size	Age	Abnormal results	Reference
Multiple symptoms	Total *N* = 700	Median (range)	Frequent reflux and/or SI ≥50%	(19)
Pulmonary	*N* = 329	2 years (1 month–16 years)	N 133 (40%)	
Gastrointestinal	*N* = 325	6.5 years (1 month–16 years)	N 114 (35%)	
Neurologic	*N* = 46	0.5 years (3 weeks–15 years)	N 23 (50%)	
Multiple symptoms	Total *N* = 181	Median (IQR): 60 days (34–108 days)	Positive SI and/or SAP Overall N 113 (62%)	(24)
Irritability	*N* = 65		N 21 (32%)	
Bradycardia	*N* = 59		N 10 (17%)	
Arching	*N* = 57		N 18 (31%)	
Cardiorespiratory events (Desaturation/bradycardia)	*N* = 47	Median (IQR): 36 days (24–66)	Positive SAP N 5 (10.6%)	(52)
Chronic cough	*N* = 426	Median (IQR): 12 months (6–39.5 months)	SAP >95% N 59 (13.8%)	(30)

**Figure 2:**
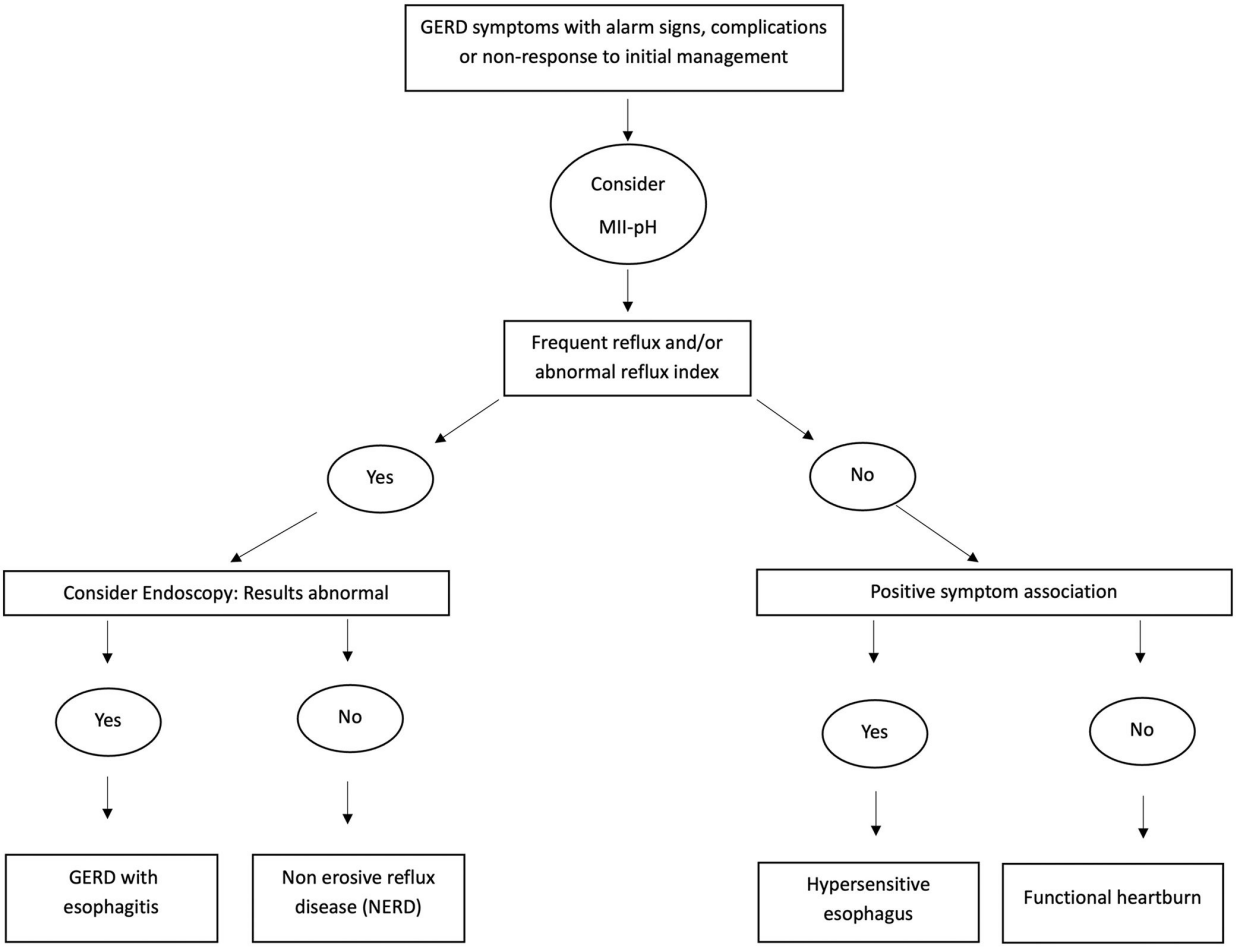
GERD phenotype classification flowchart.

Not all children with positive symptom association have pathological reflux (frequent reflux or high RI). Using both the diagnostic parameters described above in Table 2 with positive or negative symptom association, GERD may be described in terms of various disease phenotypes, as shown in Figure 2. This classification is taken from the most recent pediatric GER consensus guideline that suggests that MII-pH can be used to categorize patients into non-erosive reflux disease (NERD, abnormal MII-pH with normal endoscopy), acid GER (high RI ± frequent reflux), nonacid GER (frequent reflux + normal RI), hypersensitive esophagus or reflux hypersensitivity (infrequent reflux + normal RI but positive symptom association) and functional heartburn (normal MII-pH but ongoing symptoms) (1). A large multicenter study in children between 5 and 17 years of age showed that functional heartburn was the most common variant of GER (38%), followed by non-erosive reflux disease (NERD-26%) and acid reflux hypersensitivity (20%). The study also showed that these older children with NERD were more likely to show resolution of symptoms with acid suppression, compared to the other phenotypes (38). Although widely used in adults and older children, this disease classification is yet to be validated in younger children less than five years of age.

## 5 Utility of MII-pH

i. To define GER phenotypes as described above and guide therapy decisions (1, 17, 38–40).
ii. To study the temporal association of symptoms with GER events.
iii. To determine success of acid suppression therapy, once initiated, by repeating the MII-pH study once optimized on medication.
iv. To identify nonacid reflux and high reflux episodes, especially in patients with persistent, non-gastrointestinal or post prandial symptoms.
v. To study esophageal motility by analysis of the MII-pH waveforms and bolus transit time.
vi. To predict the presence of esophagitis using baseline impedance in the distal-most impedance channel.

The ability of MII-pH testing to evaluate for the above clinic-pathologic entities makes it particularly advantageous over other testing techniques used for diagnosis of GERD. The primary disadvantage of MII-pH compared to barium contrast studies, ultrasound and manometric studies is the lack of definitive information on variations in anatomy and esophageal motility leading to GERD. Of note, a review of symptomatic children referred for evaluation of GERD found that MII-pH detected GER in a higher proportion of patients (62% of infants and 42% of older children) compared to pH testing alone (32%), barium studies (25%), esophagogastroduodenoscopy (45%) and pepsin assay (48%) (41).

MII-pH may allow for individualized treatment in GERD with targeted use of acid suppression for children with frequent acidic reflux and reflux hypersensitivity, and neuromodulator therapy for children with functional heartburn (1). Recent studies have demonstrated the use of MII-pH to guide management decisions in symptomatic children, such as changes in medication as above, changes in diet, or referrals for anti-reflux surgery (29, 42). MII-pH can also be used to study the effect of non-pharmacological interventions to treat reflux, including left lateral positioning and thickeners (43, 44). However, unlike in adult studies, MII-pH results in children have not been shown to affect clinical outcomes such as quality of life, hospitalization rates, rates of fundoplication surgery and surgical outcomes (29, 45–47). It is often suggested that success of acid suppression therapy (AST) can be determined by follow up MII-pH studies following medication initiation. However, reflux characteristics were found to be similar in cohorts of children on and off AST, thus endorsing the need for thoughtful use of acid suppression medication for a limited duration in young children (29, 42, 48). The combined use of MII-pH with other diagnostic modalities such as video fluoroscopy, high resolution manometry and scintigraphy may be possible, to aid in the evaluation of complex dysphagia (6). Special considerations to be made when using MII-pH in patients with underlying co-morbid disease states like congenital anomalies of the esophagus, children with neurological impairment and various lung disease states have been recently described (8).

## 6 Limitations and future directions of MII-pH in young children

MII-pH is not yet widely used in children due to the lack of access to the device and the requisite training to carry out the test and to interpret results. The study, most often completed in an inpatient setting for young children, can be time-consuming and costly. Studies have shown variable degrees of interobserver agreement in the interpretation of results, with the suggestion that standardizing interpretation techniques and/or utilizing automatic analysis may improve the accuracy of the results (6, 49, 50). The current device set up may be modified to include standardized video recording technology to improve symptom yield during the study. Artificial intelligence systems may also be used to create better software algorithms to interpret patient studies faster and with greater ease and accuracy. Additionally, generative software applied to pooled patient data may be used to characterize impedance patterns in different disease processes and gain a better understanding of GERD.

The thresholds used for diagnostic parameters and symptom indices to classify GERD in MII-pH studies are difficult to validate due to a lack of data on age-specific normative values in truly healthy cohorts of young children. Further research is warranted to study symptom association using MII-pH to define the optimum time window for different symptoms in young children, and to establish causality of symptoms by testing for improvement in symptom indices after treatment. More studies are also needed to further define the association of esophageal impedance and esophageal motility and inflammation in young children, both in health and disease.

MII-pH may have additional applications in cohorts of patients with high morbidity associated with GERD, in whom treatment decisions can be challenging – such as young infants with bronchopulmonary dysplasia and other chronic lung disease states, neurologically impaired children who are at risk for aspiration, infants with complex dysphagia and those with congenital malformations of the esophagus such as tracheoesophageal fistula, especially post-surgical repair. Larger prospective studies are needed to study the impact of clinical decision-making using the results of MII-pH studies on meaningful patient outcomes in these young children such as improvement in symptom burden, improvement in lung disease and better feeding and growth patterns.

In conclusion, MII-pH is a promising tool that can be used to characterize GER in young children, study GERD symptom association and provide targeted therapies. More widespread use of MII-pH in this population will help improve our understanding of reflux in health and disease in this vulnerable population.
